# A systematic comparison of error correction enzymes by next-generation sequencing

**DOI:** 10.1093/nar/gkx691

**Published:** 2017-08-01

**Authors:** Nathan B. Lubock, Di Zhang, Angus M. Sidore, George M. Church, Sriram Kosuri

**Affiliations:** 1 Department of Chemistry and Biochemistry, University of California, Los Angeles, Los Angeles, CA, USA; 2 UCLA-DOE Institute for Genomics and Proteomics, Los Angeles, CA 90095, USA; 3 Molecular Biology Institute, University of California, Los Angeles, CA 90095, USA; 4 Genomics and Computational Biology Graduate Group, Perelman School of Medicine, University of Pennsylvania, Philadelphia, PA 19104, USA; 5 Department of Chemical and Biomolecular Engineering, University of California, Los Angeles, Los Angeles, CA 90095, USA; 6 Wyss Institute for Biologically Inspired Engineering, Boston, MA 02115, USA; 7 Department of Genetics, Harvard Medical School, Boston, MA 02115, USA

## Abstract

Gene synthesis, the process of assembling gene-length fragments from shorter groups of oligonucleotides (oligos), is becoming an increasingly important tool in molecular and synthetic biology. The length, quality and cost of gene synthesis are limited by errors produced during oligo synthesis and subsequent assembly. Enzymatic error correction methods are cost-effective means to ameliorate errors in gene synthesis. Previous analyses of these methods relied on cloning and Sanger sequencing to evaluate their efficiencies, limiting quantitative assessment. Here, we develop a method to quantify errors in synthetic DNA by next-generation sequencing. We analyzed errors in model gene assemblies and systematically compared six different error correction enzymes across 11 conditions. We find that ErrASE and T7 Endonuclease I are the most effective at decreasing average error rates (up to 5.8-fold relative to the input), whereas MutS is the best for increasing the number of perfect assemblies (up to 25.2-fold). We are able to quantify differential specificities such as ErrASE preferentially corrects C/G transversions whereas T7 Endonuclease I preferentially corrects A/T transversions. More generally, this experimental and computational pipeline is a fast, scalable and extensible way to analyze errors in gene assemblies, to profile error correction methods, and to benchmark DNA synthesis methods.

## INTRODUCTION

Synthetic DNA is a central tool for biological research (1). Notably, the initial development of nucleic acid synthesis led directly to the cracking of the genetic code (2). Today, progress in biology is often limited by the difficulty in producing long, high-quality synthetic DNA (3,4). This bottleneck is particularly apparent in the assembly of gene-sized fragments of DNA known as gene synthesis (5).

Currently, gene synthesis relies on the assembly of many oligonucleotides (oligos) of $ \sim$40–150 nucleotide (nt) into a single larger piece of DNA of $ >$1000 base-pairs (bp) (5). A variety of methods to assemble oligos into gene-sized fragments exist, but ligation- and polymerase-based assembly methods are the most common (6–9). Regardless of the method, the quality of the final product is largely dependent on the quality of the oligos used in the assembly.

Oligos are primarily synthesized using phosphoramidite chemistry first developed by Beaucage and Caruthers in the 1980s (10). Although these oligos are of high enough quality for common applications such as polymerase chain reaction (PCR), their error rates make practical gene synthesis challenging. Several groups have managed to synthesize genes from such oligos, but only find about 5–60% perfect products depending on the size and complexity of the template (11–14). This problem is further exacerbated when using lower-cost, but often lower quality oligos from array-based synthesis approaches (15–20).

Consequently, researchers have developed a number of methods to ameliorate oligo error rate post-synthesis. Size selection methods such as high-performance liquid chromatography (HPLC) or polyacrylamide gel electrophoresis (PAGE) can filter truncated sequences, but are labor-intensive and ineffective against small errors such as single-base deletions, insertions or substitutions (21,22). Hybridization-selection techniques can filter large pools of oligos, but are cost-prohibitive as the number of oligos needed effectively doubles (16,23). Sequencing-based retrieval methods can physically pick perfect sequences or separate them by barcoded PCR, but are time-intensive and can require specialized equipment (24–26). Enzymatic error correction is a more commonly used technique that is relatively inexpensive and effective against most errors. This method employs a variety of different enzymes traditionally used for mutation detection to filter out incorrect sequences by binding to or cutting at errors (27–30).

Two particular classes of proteins are most prevalent in error correction: mismatch binding proteins and mismatch cleaving proteins. Generally, these enzymes recognize distortions in the DNA helix that are caused by mishybridized bases on either strand. In gene synthesis, a pool of perfect and imperfect sequences are melted and re-annealed pairing perfect and imperfect strands to one another. This produces mishybridized bases that can be recognized by these enzymes. Mismatch binding proteins are used to enrich perfect sequences, while mismatch cleaving proteins are used (often in conjunction with exonuclease trimming) to remove imperfect sequences. The most commonly used mismatch binding protein, MutS, recognizes and binds to all single-base mismatches and a variety of small single stranded loops caused by insertions or deletions (indels) with varying affinity (31–35). There are a number of different ways to bind and separate error-containing DNA with MutS including: gel-shift assays, MutS-functionalized columns and MutS-functionalized magnetic beads (11,20,36). Mismatch cleaving enzymes operate by cutting at or near an error and a variety of different mismatch cleaving enzymes are commonly used (37). Broadly, these enzymes can correct errors in two different ways. Mismatch cleaving enzymes cut at or near errors, enabling perfect sequences to be recovered by filtration, similar to mismatch binding methods. Alternatively, exonuclease activity is used to trim the error-containing region left over by the mismatch cleaving enzymes. The full-length sequences are then recovered by performing a PCR assembly with the trimmed sequences.

Previous assessments of different enzymatic error correction methods have relied on Sanger sequencing of finished gene synthesis products to determine their efficiencies (11,12,14,19,20). These studies find that, broadly, the dominant mode of errors in gene synthesis products is single-base deletions and mismatches. However, the prohibitive cost of Sanger sequencing hundreds of thousands of bases has limited the effective characterization and comparison of existing methods. Alternatively, one can turn to the mutation detection literature to find biochemical characterizations of enzymes commonly used in error correction (30,34,38–40). Although these reports provide more detailed affinity data, they typically rely on electrophoretic methods and are thus similarly limited in sample size.

In order to overcome these limitations, we developed a custom experimental and computational pipeline that leverages next-generation sequencing (NGS) to characterize error rates. Here, we report the first in-depth characterization via NGS of both the errors arising from the assembly process, as well as the ability of six of the most commonly used error correction enzymes to eliminate these errors across 11 total conditions. With sample sizes three to four orders of magnitude larger than previous reports, we are able to gain detailed insights into the modality of errors as well as each enzyme's relative ability to correct them. We also used our method to assess the effect of polymerase on assembly quality by comparing a high-fidelity polymerase (Q5) to a low-fidelity one (KAPA2G Robust). We believe that our method can act as a generalizable platform to rapidly and cost-effectively test, characterize and optimize oligo synthesis parameters or new enzymatic error correction methods.

## MATERIALS AND METHODS

### Pre-processing

To ensure that we only analyzed high quality reads, we first ran our sequencing data through a pre-processing pipeline. First, we used BBDuk (part of the BBMap suite; version 36.14) to trim any Illumina adapters from our reads (41). Next, we used BBDuk to remove any reads with at least 26 bases that match to the PhiX (NC_001422) or *Escherichia coli* (U00096.3) genomes. We also removed any read pairs that had an ‘N’ base call in either one of the reads during this step. We then took the filtered reads and merged read pairs with perfectly overlapping regions with BBMerge (also part of the BBMap suite; version 36.14) using the pfilter = 1 option.

### Alignment and parsing

After read pre-processing and merging, we use a custom Python script to align our reads to the reference oligo sequence and parse the resulting alignments to get the positions of all errors. Our Python script uses the uta-align (version 0.1.6) package from the Python Package Index (PyPI) to perform a Needleman–Wunsch exhaustive global alignment of the input reads to the reference sequence (42). Our script can also provide functionality for performing any alignment supported by the uta-align library (e.g. Smith–Waterman local alignments), and allows for tunable gap penalties or match scores.

Once the alignment and parsing is complete, our script will output the results in a tidy csv file with the name of the read, the position of the error, the type of error and the actual error itself (43). The types of errors are as follows: M - Mismatch, D - single-base Deletion, I - single-base Insertion, P - multiPle-base deletion and S - multiple-base inSertion. The errors are classified as: (original base) (mutated base) for mismatches; the reference base(s) that were deleted for deletions; and the base(s) that were inserted for insertions. Both single and multiple-base insertions are mapped to the ‘right’ of the base in the reference sequence. For example, if the reference sequence was ‘GATTACA’ and we inserted a C at position 3, the resulting alignment can be visualized as:

**Table:** 

Position:	123-4567
Reference:	GAT-TACA
Read:	GATCTACA
CSV:	Read_1, 3, I, C

Lastly, if there is a single-base deletion or insertion in a region where there is an identical base adjacent to the mapped position of the error, we distribute the fractional count of the total number of identical bases over each position. For example, if our alignment produced a deletion of A at position 2 in the sequence ‘TAAAG,’ our software will note this as a deletion of A at positions 2, 3 and 4, with fractional counts of $1/3$ at each of those positions. This compensates for the fact that there are three equally valid alignments in that region.

### Error frequency calculations and definitions

To be consistent with previous studies, we calculated the relative error frequency per kb ($f$) as:

\begin{equation*}f{\rm{\ }} = \frac{{\mathop \sum \nolimits_{i = 1}^n {x_i}\frac{{1000}}{{{l_i}}}}}{n}{\rm{\ }}\end{equation*}

where, ${x_i}$ is the number of errors in read $i$, ${l_i}$ is the length of that read and $n$ is the total number of reads (12). This is distinct from error rates, which are defined as the number of errors detected at a given base, divided by the total number of sequencing reads in the sample. Error rates can be further separated by the specific error sub-type.

### Reagents

All the oligos were synthesized by Integrated DNA Technologies (IDT). The ErrASE Error Correction Kit was purchased from Novici Biotech and is now available as CorrectASE from ThermoFisher. The Surveyor Mutation Detection Kit was from Transgenomic. T4 Endonuclease VII was from Affymetrix. *Thermus aquaticus* MutS DNA mismatch repair protein was from Excellgen. Endonuclease V, T7 Endonuclease I, and T7 DNA Ligase were all from New England Biolabs.

### Error-enriched oligonucleotide synthesis and template assembly

The 85-nt forward and reverse oligos contains 21-nt primer sites and 64-nt template regions, 63 of which, except for the last base, were doped with 3% errors at each position (Supplementary File 1). This doping is achieved by hand-mixing 1% of every other base into the 97% of the reference base. For example, according to the reference sequence, if a position is supposed to be an A, then 1% of C, T and G was mixed into 97% A during the initial oligo synthesis by IDT. With 28 nt complementary regions, the two oligos were able to anneal and then assembled into a 142-bp doubled-stranded template. This template consists of two 21-bp primer regions and a 100 bp region for error correction and for subsequent NGS.

Specifically, to pre-assemble the forward and reverse oligos, 10.4 µl nuclease-free water (Ambion), 4 µl 5× HF Buffer (New England Biolabs), 0.4 µl 25 mM dNTP (New England Biolabs) and 0.2 µl Phusion High Fidelity Polymerase (New England Biolabs) were added into 5 µl 1 µM mixed aforementioned forward and reverse oligos. Initially heated at 98°C for 30 s, the reaction was then cycled 15 times: at 98C for 5 s, at 70°C for 1 s, ramping down with a speed of 0.5°C/s to 50°C, at 50°C for 30 s and at 72°C for 20 s. The final extension step was at 72C for 5 min. The product after the pre-assembly step was diluted 1:10 in nuclease-free water, 2 µl of which, served as template, was added into 35.25 µl nuclease-free water, 10 µl 5× HF Buffer, 1 µl 25 mM dNTP, 0.5 µl Phusion High Fidelity Polymerase, 1.25 µl 10 mM mixture of forward and reverse PCR amplification primers to make the total volume of this PCR 50 µl (Supplementary File 1). Initially heated at 98°C for 30 s, the reaction was then cycled 25 times: at 98°C for 5 s, at 62°C for 10 s, at 72°C for 10 s. The final elongation step was at 72°C for 5 min. Pooled PCR products were then cleaned using QIAquick PCR Purification Kit (Qiagen), and the purified products served as the template for subsequent error correction treatments and sequencing.

### Error correction of the synthetic DNA template

#### ErrASE

Per the manufacturer's instructions, 60 µl of $ \sim$50 ng/µl template in 1× HF Buffer was re-annealed to form heteroduplex by heating at 98°C for 1 min, cooling at 0°C for 5 min and incubating at 37°C for 5 min. Next, 10 µl of this re-annealed heteroduplex was added into each well of the 6-well ErrASE tube and was incubated at room temperature for 1 h. We then combined 2 µl from each well as template into the recovery PCR, whose setup and thermocycling conditions were the same as the assembly PCR in the section above. The PCR product using the treated heteroduplex from the first well of the ErrASE tube (presumably has the highest concentration of ErrASE) presented a band, indicating successful recovery after error correction. This product was thus cleaned-up using QIAquick PCR Purification Kit and served as the template for the second iteration of ErrASE treatment.

#### Surveyor

Per the manufacturer’s instructions, $ \sim$50 ng/µl template in 1× HF Buffer was re-annealed to form heteroduplex by the following thermocycling conditions. First, the sample was heated at 95°C for 10 min. Then, the temperature was ramped down at 2°C/s, and was held at 85°C for 1 min. Finally, the temperature was further cooled down to 25°C at 0.3°C/s, and was held for 1 min at every 10°C interval. Per Saaem *et al.*, 2 µl Surveyor Nuclease S and 1 µl Enhancer S were added into 8 µl re-annealed heteroduplex (19). The reaction mixture was then incubated at 42°C for 60 min. After the treatment was concluded, 2 µl of the mixture served as the template in the recovery PCR, whose setup and thermocycling conditions were the same as the assembly PCR. The product of this recovery PCR, once cleaned-up, entered the next round of Surveyor Nuclease treatment.

#### Endonuclease V

Similar to Fuhrmann *et al.*, 10 µl of $ \sim$50 ng/µl template in 1× HF Buffer was re-annealed using the cycling condition described in the ErrASE section (12). We then added 5U of Endonuclease V, 2 µl of NEBuffer 4 and nuclease-free water to the re-annealed heteroduplex to make the total volume 20 µl. The reaction was incubated at 37°C for 24 h, and 2 µl of this mixture served as the template for the recovery PCR. The cleaned-up product then entered the next iteration of Endonuclease V treatment.T7 Endonuclease I (Fuhrmann).

As in Fuhrmann *et al.*, 10 µl of $ \sim$50 ng/µl template in 1× HF Buffer was re-annealed using the cycling condition described in the ErrASE section (12). We combined 2 µl of NEBuffer 2, 25U of T7 Endonuclease I and nuclease-free water to make the final volume 20 µl. The reaction was incubated at 37°C for 24 h, and 2 µl of the mixture served as the template for the recovery PCR. The cleaned-up product entered the next iteration of T7 Endonuclease I treatment.

#### T7 Endonuclease I with T7 DNA Ligase

We first re-annealed 100 ng of template in 1× HF Buffer according to the ErrASE protocol. Then we combined 2.5 µl of T4 DNA Ligase reaction buffer, 10U of T7 Endonuclease I, T7 DNA Ligase (at 0, 1000U or 10000U) and the appropriate amount of nuclease-free water to make the final volume 25 µl. The reaction was then incubated at 25°C for 4 h, and 2 µl of the treated sample served as the template for recovery PCR. We used 100 ng of the cleaned-up product for the next iteration of T7 Endonuclease I/T7 DNA Ligase treatment.

#### T4 Endonuclease VII

First, 10 µl of $ \sim$50 ng/µl template in 1× HF Buffer was re-annealed using the cycling condition described in the ErrASE section. Then, 1 µl 1M Tris–HCl (pH 8.0), 4 µl 50 mM MgCl_2_, 2 µl 100 mM $\beta$-mercaptoethanol, 1 µl 10 mg/ml bovine serum albumin and 2 µl T4 Endo VII (1000U) was added to the 10 µl heteroduplex. The reaction mixture was incubated at 37°C for 24 h, and 2 µl of which served as the template for the recovery PCR. Then the cleaned-up PCR product entered the next cycle of T4 Endonuclease VII.

#### MutS

Per the manufacturer’s instructions, 250 ng/µl in 10 mM Tris–HCl (pH = 7.8) and 50 mM MgCl_2_ was heated to 95°C for 5 min followed by cooling at 0.1°C/s to 25°C. To the re-annealed template, 207.39 µl 1× binding buffer (20 mM Tris–HCl (pH = 7.8), 10 mM NaCl, 5 mM MgCl_2_, 1 mM Dithiothreitol and 5% glycerol) was added, making the concentration of DNA template to $ \sim$11.5 ng/µl. This mixture was then aliquoted into two tubes with 109 µl in each. Appropriate amount of MutS was added into each of the tubes so that the final MutS concentration was 950 and 1900 nM, respectively. The mixtures were then incubated at room temperature for 20 min. Equal volumes of Amylose Resin (New England Biolabs), washed and pre-equilibrated with 1× binding buffer, were added into the tubes. The mixtures were incubated at room temperature for 30 min, before being spun down. We purified the supernatants with a Qiagen MinElute kit, an eluted the product in 10 µl EB. We used 2 µl of the 1:100 diluted elution as the templates for the recovery PCR. At last, we pooled the PCR products, cleaned them up and used them for the next iteration of MutS treatments.

### Next-generation sequencing using Illumina MiSeq

Each of the control and enzymatically treated samples was prepared as an individual sequencing library. In summary, the sequencing libraries were prepared using two rounds of qPCR, with the first round appending the Illumina P5 sequence and the second appending the P7 sequence as well as the indices. We also note that the KAPA SYBR FAST kit is a Taq-based polymerase. Specifically, the first round of PCR was set up by mixing 25 µl KAPA SYBR FAST Universal 2× qPCR Master Mix (KAPA Biosystems), 1 µl 10 µM Multiplexing PCR Primer 1.0, 1 µl 10 µM Multiplexing PCR Primer 2.0, 1 µl $ \sim$100 pg/µl error correction DNA template and 22 µl nuclease-free water. Per the manufacturer’s instructions, the two-step thermocycling protocol was used for the qPCR reactions. Once the signals reached the plateaus, the reactions were stopped and cleaned-up using Agencourt AMPure beads, according to the manufacturer’s instructions. The final elution volume was 30 µl. To set up the second round of PCR, 25 µl KAPA SYBR FAST Universal 2× qPCR Master Mix, 1 µl 10 µM Multiplexing PCR Primer 1.0, 1 µl 10 µM PCR Primer each with a distinct index, 1 µl $ \sim$100 pg/µl template from the first round PCR and 22 µl nuclease-free water. The thermocycling and cleaned-up procedures remained the same as those in the first round of PCR. Then, the individually prepared sequencing libraries were quantified using the Library Quantification Kit-Illumina (KAPA Biosystems), according to the provided protocol. Barcoded libraries were subsequently mixed to $ \sim$10 nM concentration, and the mixed libraries were quantified again before being loaded onto an Illumina MiSeq with a V2 300 cycle kit.

### Five-oligo assembly with high- and low-fidelity polymerases

We designed two 220-bp constructs that can be assembled from five 60-nt oligos each (Supplementary File 1). Each overlap region between adjacent oligos is 20-bp in length, and the first and last oligo contain 15-bp forward and reverse priming regions used for assembly. All overlap and priming sequences were taken from the set designed in Eroshenko *et al.* to minimize cross-hybridization and maximize ${T_m}$ similarity (44). Each set of five oligos was synthesized by Integrated DNA Technologies (IDT) with no modifications and pooled into two 1 µM five-oligo mixes.

To pre-assemble the five-oligo construct, 5 µl of each 1 µM five-oligo mix was added to 10 µl of NEBNext Q5 HotStart HiFi PCR Master Mix or KAPA2G Robust HotStart ReadyMix and 5 µl nuclease-free water. Initially heated at 98°C for 30 s, the reaction was then cycled 15 times: at 98°C for 5 s, at 70°C for 1 s, ramping down with a speed of 0.5°C/s to 50°C, at 50°C for 30 s and at 72°C for 20 s. The final extension step was at 72°C for 5 min. The product after the pre-assembly step was diluted 1:10 in nuclease-free water, 2 µl of which, served as template, was added into 20.5 µl nuclease-free water, 25 µl of Q5 or KAPA2G Robust master mixes and 1.25 µl 10 mM mixture of forward and reverse amplification primers flanking the outer oligos of each construct. Initially heated at 98°C for 30 s, the reaction was then cycled 20 times: at 98°C for 5 s, at 62°C for 10 s, at 72°C for 10 s. The final elongation step was at 72°C for 5 min. Pooled PCR products were then purified using a DNA Clean and Concentrator-5 (Zymo).

We prepared each assembly as an individual sequencing library with two technical replicates. The sequencing libraries were prepared using a single round of PCR, which appended both the Illumina P5 and P7 sequences as well as the indices. Specifically, 0.01 ng of template was added to 20.5 µl nuclease-free water, 25 µl Q5 or Robust (depending upon initial condition) and 1.25 µl 1 µM forward and reverse sequencing primer with corresponding distinct indices. Each library was amplified for a small number of cycles (∼12–14) empirically determined using KAPA SYBR FAST Universal 2× qPCR Master Mix (KAPA Biosystems). We estimate the the total number of amplification cycles to <50 (<15 for pre-amplification, 20 for amplification, 12–14 for NGS prep). Individually prepared sequencing libraries were quantified using an Agilent TapeStation 2200. Barcoded libraries were subsequently pooled and mixed to 20 nM concentration, and prepared for sequencing on an Illumina MiSeq with a 500-cycle V2 kit.

## RESULTS

### Next-generation sequencing based analysis of a model gene assembly

**Figure 1.**
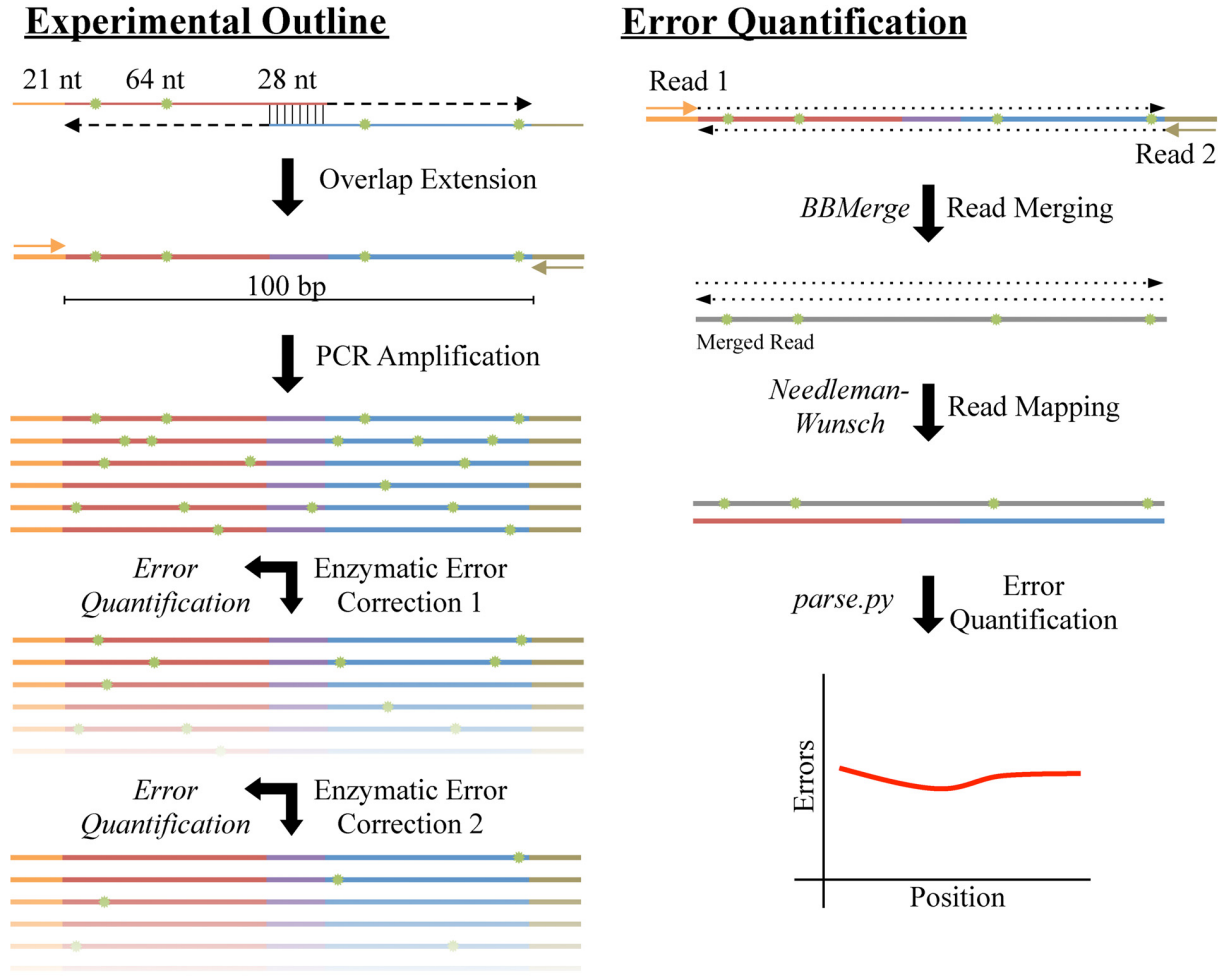
Schematic of enzymatic error correction and downstream data processing. We assembled our 142 bp product from two 113 nt oligos consisting of a 21 nt primer, a 64 nt payload and a 28 nt overlap region. After annealing and overlap extension, we amplified our template via polymerase chain reaction (PCR), yielding 100 bp of template in-between the primer sites. We then denatured and re-annealed the PCR products to form heteroduplexes, thereby exposing any errors (shown in green). After, we subjected the pool of heteroduplexes to two successive rounds of ten different enzymatic error correction treatments. At each step, we took aliquots and sequenced the products on an Illumina MiSeq with fully overlapping forward and reverse reads. To mitigate sequencing errors, we used BBMerge to merge reads with a perfect agreement between the forward and reverse reads. We then aligned these sequences to the designed reference using an exhaustive Neeleman–Wunsch aligner to minimize alignment artifacts. Finally, we further processed the alignments to quantitate the types and extent of different errors across all conditions.

To assess different enzymatic error correction methods, we first constructed a constant reference sequence that served as the base for downstream analyses. We designed this sequence to have a length of 100 bp (not including two 21 bp priming regions for amplification and sequencing), a balanced nucleotide content (26:23:23:28 A:C:G:T content), good coverage of all nucleotide pairs and most triplets (80%) while limiting homo-polymer repeats greater than two, and a 28-bp region in the center that has good melting temperature and low secondary structure to facilitate overlap-extension assembly of the two primers. We assembled this sequence from two 85 nt oligos by a preliminary round of polymerase chain assembly. We then diluted the products of that reaction and used PCR to amplify the full-length 142 bp construct (Figure 1). We then subject the resulting assembly to multiple rounds of enzymatic error correction and sequence the products at each step.

We expect that errors arising during sequencing will convolute our true signal. In order to limit these errors as much as possible, we developed a stringent data processing pipeline briefly outlined as follows: first, we cleaned our raw sequencing reads (509 717 per sample on average) by trimming sequencing adapters, removing any reads containing ‘N’ base calls (212 reads on average) and filtering out any reads that aligned to either the PhiX or *E. coli* genomes with BBDuk (822 reads on average). This ensures that any spurious reads will not contaminate our alignments and lead to false-positive error calls. Next, we merged our paired end reads together with BBMerge, only keeping alignments with perfect correspondence between the forward and reverse reads. Since we sequenced our assembly with fully overlapping reads, each base is effectively sequenced twice. We found that an average of 95.2% of all bases in the merged reads had a Phred33 score (Q) of 41 ($ \sim$1/12600 chance of being miscalled), and 99.8% of all bases on average were above Q30 (1/1000 chance of being miscalled). It should also be noted that most bases were probably above Q41 as this is the default maximum Phred score for most read mergers to maintain backward compatibility with legacy software. The merging step removed an average of 15.8% of input reads, resulting in an average of 426 514 reads per sample at the end of processing.

After pre-processing the reads, we used a Python implementation of the Needleman–Wunsch aligner, uta-align, to align our reads to the perfect reference sequence. We elected to use a Needleman–Wunsch aligner as it is guaranteed to converge on the optimal alignment for a given scoring system (45,46). In contrast, typical short read aligners such as BWA and Bowtie2 do not offer such guarantees as they use heuristics to trade accuracy for speed (47,48). We find that these heuristics often result in sub-optimal alignments and miscategorization of error sub-types (Supplementary Figure S1 and Table S1).

### Error-doped oligos enable comparisons

In order to assess the sensitivity of our assay, we treated our two-oligo assembly with the error correction cocktail ErrASE and measured the resulting error rates (Supplementary Figure S2). Although we were able to measure significant (Mann–Whitney U, $P < < 0.001$, Holm-corrected) reductions in the rate of single-base deletions, multiple-base deletions and single-base insertions, we were not able to find a significant (Mann–Whitney U, NS, Holm-corrected) reduction between the median rate of mismatches. To ensure that we had a measurable change in error rates for mismatches after enzymatic treatment, we assembled our template from oligos that had errors doped into the sequence. Specifically, we ordered each base with 97% of the intended base, and 1% of the other 3 nt (not including the 21 bp priming region and the last base of the oligo).

**Figure 2.**
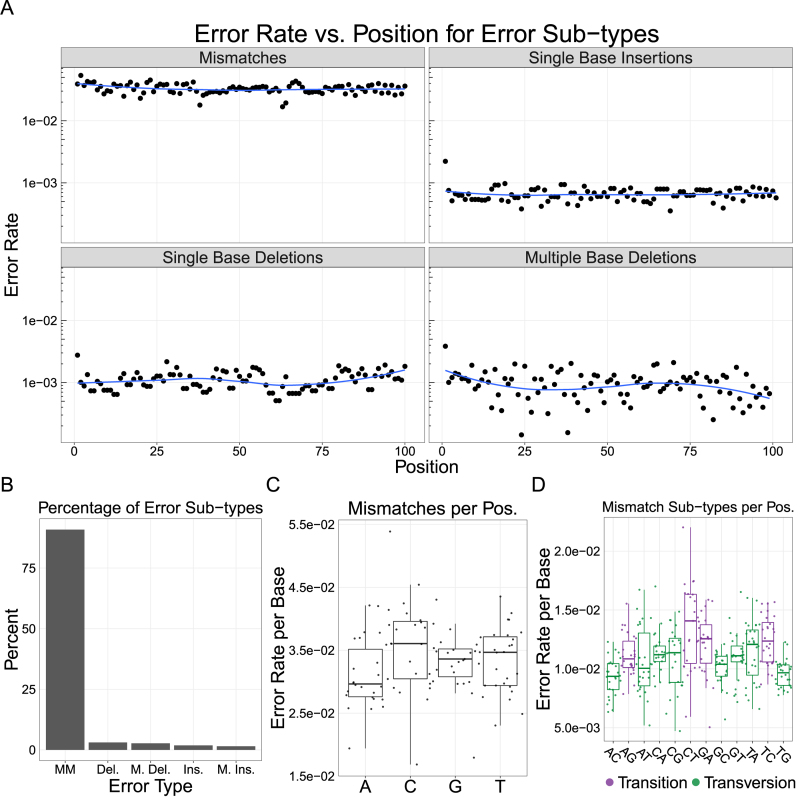
Analysis of error rates in a model gene assembly created from error-doped oliogs. (**A**) The error rates per base are plotted across each position in our model separated by the four major classes of error types. We do not see strong positional effects for errors across the template. (**B**) We find a majority of errors on the template are mismatches (MM), followed by single (Del.) and multiple base (M. Del.) deletions; Single (Ins.) and multiple base (M. Ins.) insertions occur at even lower frequencies. (**C**) There are no significant differences between the median rate of mismatches at any base (Mann–Whitney U, NS). (**D**) Similarly, there are no significant differences between transitions and transversions (Mann–Whitney U, NS), implying that the errors were doped uniformly into our oligos. Note: blue line is a LOESS fit; box plots are first and third quartile for hinges, median for bar and 1.5 × the inter-quartile range for whiskers.

We found that the errors were doped uniformly into our assembly (Figure 2A), with the majority of errors being mismatches (90.9%), followed by single base deletions (3.1%), multiple base deletions (2.7%), single base insertions (1.9%) and multiple base insertions (1.5%; Figure 2B). Unlike the standard oligo assembly (Supplementary Figure S3), we found no significant difference between the median mismatch rate ($3.99\ \times {10^{ - 2}}$) at any of the four bases (Mann–Whitney U, NS; Figure 2C). Similarly, the median rate of individual transitions and transversions were not significantly different from each other (Mann–Whitney U, NS; Figure 2D). These data suggest that incorrect bases were doped in to our oligos at an approximately equal rate that exceeded the baseline error rate of KAPA SYBR Fast—the other potential source of mismatches. We note that the median rates of all error types were significantly higher in the error-doped assembly than in the standard assembly (Supplementary Table S2 and Figure S4; Mann–Whitney U, $P < < 0.001$). Although this is expected for mismatches, we suspect that the higher median error rates for the other error sub-types are a result of the non-standard synthesis required to dope the errors into our oligos.

### Enzymatic error correction improves assembly quality

**Figure 3.**
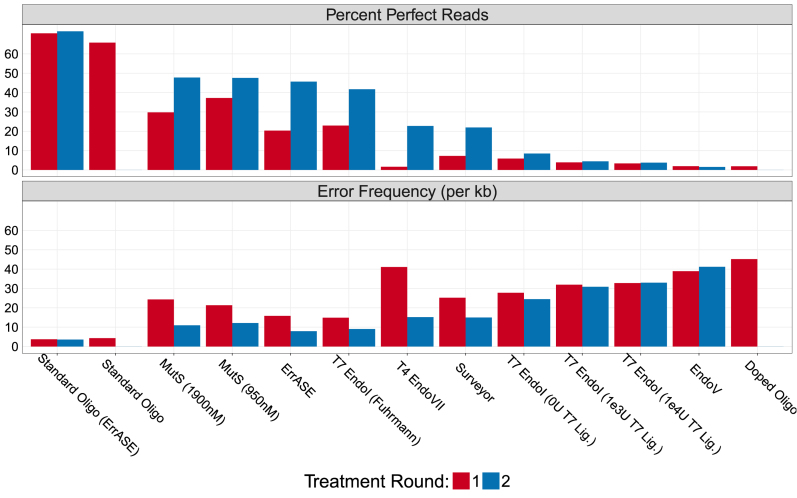
Effectiveness of enzymatic error correction methods. Here, we compare the error frequency (errors/kb) and number of perfect assemblies for 10 different enzymatic error correction methods. We find that MutS is the most effective enzyme at increasing the percentage of perfect assemblies. However, ErrASE is the most effective at decreasing error frequency. Additionally, we see that the efficacy of T7 Endonuclease I and MutS are dependent on protocol, and that the addition of a ligase had detrimental effects on sequence quality. Note: the *x*-axis is ordered by decreasing number of perfect assemblies.

Having established the error profile of the error-doped assembly, we evaluated 10 different enzymatic error correction methods using six different enzymes on their ability improve the quality of this assembly (Figure 3). As expected, consecutive rounds of enzymatic error correction improved both the relative error frequencies and the number of perfect assemblies. ErrASE was the most effective at decreasing the error frequency, with two rounds of treatment dropping the error frequency from the doped oligo rate of 45.1 to 7.9 errors/kb. The next most effective enzyme at decreasing error frequency was T7 Endonuclease I (9.1 errors/kb). Based on previous reports in the mutation detection literature, we hypothesized that the addition of a ligase with T7 Endonucluase I would improve correction (39). We find that the addition of T7 ligase actually decreased assembly quality relative to the no ligase control. In agreement with previous studies, we also find that T7 Endonuclase I is highly sensitive to protocol and concentration as exhibited by the wide range of error frequencies (12,14). After T7 Endonuclease I, we found MutS to be the third most effective enzyme at 10.9 errors/kb, with T4 Endonuclease VII, Surveyor and Endonuclease V following.

However, when looking at number of perfect assemblies sequences, MutS was the most effective enzyme treatment. MutS increased the percentage of perfect sequences in the doped oligo from 1.9 to 47.8% (47.6% for 950 nM), while ErrASE increased it to 45.6%, and T7 Endonuclease I increased it to 41.7%. In other words, the oligos that are imperfect after the MutS treatment have more errors on average than those after the T7 Endonuclease I and ErrASE treatments.

### Differences in enzymatic error correction

**Figure 4.**
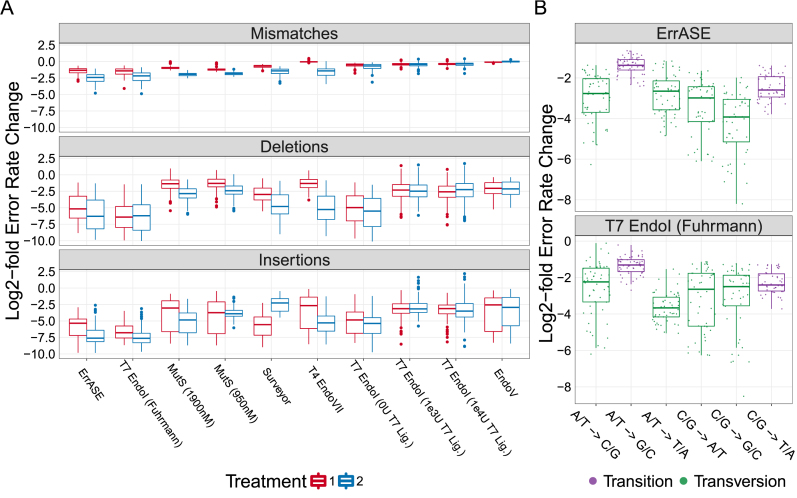
Relative decrease of different error types. (**A**) All enzymes were able to correct both single- and multiple-base insertions and deletions. Additionally, we find that the best performing enzymes corrected the highest amount of mismatches. Note: the *x*-axis is ordered by increasing error frequency. (**B**) We measure significant differences between the median decrease in C/G → G/C mismatches and the bulk median of all other mismatches after two treatments of ErrASE. Similarly, two treatments of T7 Endonuclease I results in a significant difference between the median decrease in A/T → T/A mismatches compared to the bulk median of all other mismatches (both Mann–Whitney U, *P* << 0.001).

With an average of 426 514 reads per round of error correction, our method provides sample sizes three to four orders of magnitude higher than any previous study. This enabled us to compare the effectiveness of these enzymes on rarer errors such as insertions that would be inadequately sampled with Sanger sequencing. Using the error-doped template as a reference, we measured the relative change in error rates for each position across all different enzymatic error correction methods (Figure 4A).

We see that in general, all enzymes tested were able to correct insertions and deletions. We find that enzyme performance (as measured by error frequency or number of perfect assemblies) is directly related to the ability to correct mismatches. For example the best performing enzymes, ErrASE, T7 Endonuclease I and MutS, were able to decrease the median mismatch error rate relative to the error-doped input by 6.2-, 5.1- and 4.2-fold, respectively. In contrast, the worst performing enzyme, Endonuclease V, was unable to decrease the median mismatch error rate relative to the error-doped input.

We next sought to measure differences in affinity for specific errors between enzymes (Supplementary Figures S5–7). We were unable to measure any significant differences between bases for the median fold reduction of insertions and deletions (Kruskal–Wallis, NS) across all enzymes after two treatments. However, we were able to detect significant differences between the median fold reduction of different mismatches (Kruskal–Wallis, $P < < 0.001$) across all enzymes after two treatments. Based on these data, we searched for specific mismatch correction biases in our best performing enzymes (Figure 4B). For example, we found that two rounds of ErrASE or MutS treatment resulted in a significantly different change in the median fold reduction of C/G $ \to$ G/C mismatches as compared to the bulk median of all other mismatches (15.2- versus 5.4-fold for ErrASE; 5.1- versus 4.1-fold for MutS; Mann–Whitney U, $P < < 0.001$). In contrast, two rounds T7 Endonuclease I did not result in significant changes in the median fold reduction of C/G $ \to$ G/C mismatches (5.6- versus 5.1-fold; Mann–Whitney U, NS). They did however, significantly change the median fold reduction of A/T $ \to$ T/A mismatches as compared to the bulk median of all other mismatches (12.7- versus 4.2-fold; Mann–Whitney U, $P < < 0.001$).

Taken together, these data suggest that different enzymatic error correction methods could be used for different applications. For example, GC- or AT-rich constructs would be best corrected by ErrASE and T7 Endonuclease I, respectively. Alternatively, MutS can be used for applications such as protein libraries, where the proportion of perfect sequences are paramount. We also note that the relative rate of correction for transitions and mismatches in general is likely lower than what is measured here due to errors incorporated by the Taq-based KAPA SYBR Fast polymerase after during the NGS preparation (49–54). For example, the median fold correction of A/T $ \to$ G/C transitions (the most common Taq-based error) was significantly different than that of the bulk median for all other mismatches for ErrASE, MutS and T7 Endonuclase I (2.6- versus 7.1-fold for ErrASE; 2.8- versus 4.4-fold for MutS; 2.5- versus 6.8-fold for T7 Endonuclease I; Mann–Whitney U, $P < < 0.001$).

### Analysis of two five-oligo assemblies

In order to investigate the effect of polymerase fidelity on assembly quality, as well as the performance of our method on longer constructs, we assembled two 220-bp constructs from five 60-nt oligos with 20 bp overlaps. To facilitate annealing, we designed the overlap regions to have ∼50% GC content and minimal secondary structure. We used random nucleotide sequences between the overlap regions with the single restriction being no single nucleotide repeats longer than four. The resulting nucleotide content of the two constructs are relatively balanced (47:50:62:61 - A:C:G:T for construct one, and 52:53:58:57 - A:C:G:T for construct two). We assembled both constructs with either Q5 or KAPA2G Robust polymerases, and sequenced the assemblies in duplicate with an Illumina MiSeq (∼242 000 reads per sample on average after the pipeline filtering). Technical replicates show high correspondence (Supplementary Figure S8) and the error profiles were consistent for each polymerase across the two constructs (Supplementary Figure S9).

**Figure 5.**
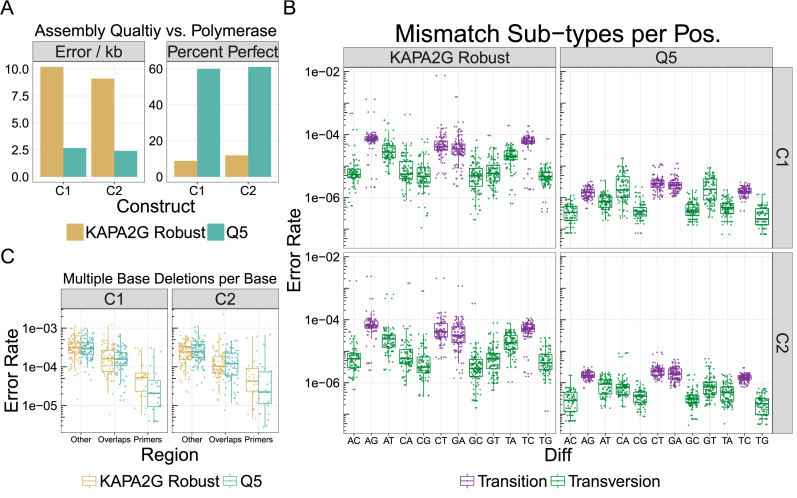
Effect of polymerase on assembly quality. We assembled two different 220 bp constructs (C1 and C2) from five 60 nt oligos with 20 bp overlaps with Q5 and KAPA2G Robust polymerases. (**A**) We find that the average error frequency of both constructs is significantly higher for KAPA2G Robust than for Q5 (9.7 versus 2.5 errors/kb). We observe similar trends for the average percentage of perfect assemblies (60.5% for Q5 and 10.4% for KAPA2G Robust). (**B**) Similar to the two-oligo assembly, we find that the Taq-based KAPA2G Robust polymerase also has a higher rate of transitions than transversions (mean of $5.32\ \times \ {10^{ - 5}}$ versus $6.40\ \times \ {10^{ - 6}}$ over both constructs; Mann–Whitney U, *P* << 0.001). (**C**) We find that the median rate of multiple base deletions per base in the overlap regions decreased ∼2-fold relative to non-overlapping regions for both polymerases (Mann–Whitney U, *P* << 0.001). Similarly, the median rate of multiple base deletions per base also significantly decreases in the priming regions for both KAPA2G Robust(∼6-fold) and Q5 (∼13-fold) for both constructs (both Mann–Whitney U, *P* << 0.001). The difference in decrease between the polymerases was not significant.

As expected, constructs assembled with Q5, a high-fidelity polymerase, had lower error frequencies (2.5 versus 9.7 errors/kb) and a larger percentage of perfect constructs (60.5 versus 10.4%) than KAPA2G Robust, a Taq-based polymerase (Figure 5A). The majority of this difference is caused by the higher mismatch frequency in the KAPA2G Robust samples (Table 1). The frequencies of errors other than mismatches are very similar between the two polymerases (Table 1). These errors are likely due to oligonucleotide synthesis, as polymerase and Illumina sequencing errors are most often mismatches. Using the previously measured error rates of $ \sim 2\ \times {10^{ - 4}}$ errors/kb/cycle for Q5, we estimate the expected error frequencies of our assemblies to be $ \sim$0.01 error/kb after 50 rounds of amplification with Q5 polymerase (52). Since this value is an order of magnitude lower than our measured mismatch rate (0.21 mismatch/kb), we estimate the upper bound of mismatches in these model assemblies to be ∼0.2 mismatches/kb.

**Table 1.** Estimated error frequency for model gene assemblies

Error type	Q5	KAPA2G Robust
Mismatches	0.2131 ± 0.0019	7.1388 ± 0.0121
Single base deletions	2.0121 ± 0.0062	2.1891 ± 0.008
Single base insertions	0.0747 ± 0.0011	0.0816 ± 0.0014
Multiple base deletions	0.2326 ± 0.002	0.2342 ± 0.0029
Multiple base insertions	0.0014 ± 2e-04	0.0083 ± 4e-04

Here, we averaged the errors/kb for both five-oligo assemblies using Q5 and KAPA2G Robust polymerases and their technical replicates across each error type (errors are standard error of the mean). We see that all error subtypes are similar except for mismatches.

In agreement with our two-oligo assemblies (Supplementary Figure S3), the KAPA2G Robust amplified assemblies also had a higher median error rate per base for transitions ($5.32\ \times {10^{ - 5}}$) than for transversions ($6.39\ \times {10^{ - 6}}$) across both constructs (Mann–Whitney U, $P < < 0.001$; Figure 5B). These errors agree with previous single-molecule studies of this polymerase, and suggest that KAPA SYBR Fast was indeed incorporating mismatches during our NGS preparation for the two-oligo assembly (50,52). We note that the KAPA2G Robust assemblies had a very high mismatch rate at the bases immediately before and after the third and fifth overlaps. We did not observe this issue in assemblies of the same oligonucleotide mixtures assembled by Q5.

Next, we measured the effect of the overlapping regions on the number of multiple base deletions (Figure 5C). In congruence with our data from the two-oligo assembly, we found that the median rate of multiple base deletions (for a given position in the assembly) was significantly different in the overlap regions than in the rest of the assembly with an average reduction of $ \sim$2-fold for both Q5 and KAPA2G Robust across the constructs (Mann–Whitney U, Holm corrected; $P < < 0.001$). We found no significant decrease in the rates of single base deletions in the overlapping regions. Since we added our sequencing primers by annealing to the first and last 15 bp of the constructs, we could also measure the effect of multiple base deletions in the priming region. Again, we found that the rate of multiple base deletions in the priming region was significantly different than both the overlap region and the rest of the assembly, with an average reduction of $ \sim$13-fold for Q5 and $ \sim$6-fold for KAPA2G Robust (Mann–Whitney U, Holm corrected; $P < < 0.001$). The differences in reduction between Q5 and KAPA2G Robust were not significant, likely due to a small sample size ($n \approx 25$).

## DISCUSSION

One of the most promising methods to improve the quality of gene synthesis products is enzymatic error correction. Previous characterizations of error correction enzymes were limited by Sanger sequencing, which prohibited deep enough sequencing to adequately sample rare variants. Here, we surpass this bottleneck by leveraging NGS and a custom computational pipeline to analyze errors in multiple model gene assemblies. With sample sizes of three to four orders of magnitude greater than any previous study, we were able to accurately quantitate error frequencies and sample rare errors such as insertions. In addition, NGS precludes the need for time consuming cloning steps. This enabled us to rapidly compare six of the most commonly used error correction enzymes in a total of eleven different conditions in a single experiment, and marks the first comprehensive comparison of enzymatic error correction methods via NGS.

We took multiple steps to minimize the number of false error calls resulting from our method. First, we sequenced our assembly with fully overlapping paired-end reads. Since each base is called independently twice and we only merge reads with a perfect match between the forward and reverse reads, it is unlikely that many sequencing errors made it through this filter. We also compared the error profile of the Needleman–Wunsch alignment to two commonly used short-read aligners, BBMap and Bowtie2. As BBMap and Bowtie2 use heuristics that trade accuracy for speed, we found that their resulting alignments were sub-optimal and led to higher false error calls relative to the Needleman–Wunsch alignment.

We assessed the sensitivity of our method by comparing the error rates of a two-oligo assembly before and after ErrASE treatment. We could measure significant changes in all errors except for mismatches. We hypothesized that our Taq-based polymerase (KAPA SYBR FAST) had re-incorporated mismatches during the NGS preparation. To ensure that we could measure changes in the amount of mismatches, we re-assembled our model sequence with oligos synthesized with 3% of the incorrect base at every position. We expected that the net change in mismatches in the error-doped template after error correction would be larger than the basal error rate of the polymerase, enabling quantification. Additionally, increasing the error rate gives a more realistic number of errors (3-4) per assembly that might occur in a longer gene synthesis.

We then used our method to test the ability of six of the most common error correction enzymes in eleven total conditions to improve the quality of the error-doped assembly. As expected, we found that all error correction enzymes were able to decrease the error frequency and increase the number of perfect assemblies. We also found that two consecutive treatments of error correction were more effective than one. We then leveraged the large sample sizes generated by NGS to probe specific differences between different enzyme treatments. These data suggest that ErrASE would be the most effective at correcting GC-rich templates, and T7 Endonuclease I is the most effective at correcting AT-rich templates. Alternatively, MutS would be appropriate for the most common applications requiring a single sequenced-verified perfect assembly. The discrepancy of average error frequency and percentage of perfect sequences highlights the importance of using the metrics that are most appropriate for the given downstream application. In addition, we find that performance of these enzymatic treatments is sensitive to the protocol used as shown in the MutS and T7 Endonuclease I assays.

To test the effect of the polymerase on assembly quality, we assembled two 220 bp constructs from five oligos with both KAPA2G Robust and Q5 polymerases, and compared their error profiles. As expected, we measured a significantly higher number of mismatches in the KAPA2G Robust assemblies than in the Q5. Since the expected mismatch rate of Q5 is lower than our measured value, we estimated an approximate upper bound on the underlying error frequencies of two model gene assemblies. This is corroborated by the fact that the frequencies of all error types except for mismatches agreed between the two polymerases. Thus, the most common errors in our assemblies were single base deletions, when controlling for polymerase effects. This agrees with previous studies of enzymatic error correction (11,14,19). Two other studies found mismatches to be the most common error. In the first study, this is likely explained by the fact that they amplified their constructs with Taq-polymerase (12). The second study assembled their genes from chip-synthesized oligos, which might have different error profiles (20). Lastly, we found that the overlapping regions of our assembly were effective at decreasing the rate of multiple base deletions, but were ineffective for single base deletions.

Our method in its current iteration has limitations. For one, any polymerase misincorporations will convolute the true mismatch correction rate of a given enzyme. While we show that using a high-fidelity polymerase throughout the assembly and NGS library preparation ameliorates this issue, we might still be observing library preparation artifacts. Alternatively, we can incorporate random barcoding strategies or utilize single molecule sequencing to further eliminate polymerase errors (50,52,54). Second, Illumina sequencing limits our assessments to assemblies $ < 600$ bp. We could extend our methodology to long-read technologies such as PacBio or Oxford Nanopore to assess kilobase-scale gene synthesis products (55). At these lengths, we would likely have to switch from a Needleman–Wunsch alignment to more optimized versions to avoid a significant time penalty (56). At last, our model two-oligo assembly used to analyze enzymatic error correction is not indicative of a typical gene synthesis product as it does not code for a gene, is shorter than standard assemblies (142 bp), is assembled from only two oligos and has a contrived mismatch error rate.

Overall, our method is a fast and accurate method for looking at errors in arbitrary sequences. We believe that this method will be useful for not only rapidly profiling new enzymatic error correction methods, but for other applications such as assessing the quality of chip-synthesized oligos or developing new gene synthesis methods.

## AVAILABILITY

The computational pipeline described above is open source, free to use under the MIT license and available at https://github.com/kosurilab/errorCorrect. For the final analysis and figure production, we used R (version 3.3.*) and ggplot2 (57,58).

## ACCESSION NUMBER

Sequencing data are available from the sequencing read archive (SRA) with the accession number SRP110084.

## Supplementary Material

Supplementary Data
