# Supplementary material for: A systematic comparison of error correction enzymes by next-generation sequencing
Source: Nucleic Acids Res. 2017 Aug 1;45(15):9206–17. doi: 10.1093/nar/gkx691 (PMC5587813; doi:10.1093/nar/gkx691)
Supplement: Supplementary Data [file gkx691_Supp.zip › nar-00160-n-2017-File007.pdf]

# Supplementary Information

May 4, 2017

## Analysis of a Two Oligo Assembly

We applied our pipeline to quantify the different types of errors found in our two-oligo assembly of standard (not error doped) oligos (Figure S3). We find that on average about one-third of assemblies contain errors, with an overall error frequency of approximately 4.3 errors per kb. We find that mismatches account for the majority of errors (∼75%), followed by single (∼14%) and multiple-base deletions (∼8%) (Figure S3A). The mismatches segregate into two significantly different populations, with the median error rate per base being higher at A's ($4.33 \times 10^{-3}$) and T's ($4.25 \times 10^{-3}$) than at G's ($1.68 \times 10^{-3}$) and C's ($1.91 \times 10^{-3}$) (Figures SB, C; Mann-Whitney U, $p << 0.001$, Holm-corrected). Furthermore, we find that the median rate of transitions was significantly higher than that of transversions for each base (Figures 2C; Mann-Whitney U, $p << 0.001$, Holm-corrected). All of these observations indicate that much of the mismatch error rate is due to polymerase misincorporation during the amplification steps for assembly and sample-preparation for sequencing. Specifically, we used the Taq-based KAPA SYBR Fast polymerase during next-generation sequencing library preparation steps. Consistent with our observations, misincorporations caused by Taq occur most often at A's and T's, and are preferentially A/T → G/C transitions (49–52). However, we cannot completely rule out the effect of errors in the oligo synthesis as our error frequency of 4.3 errors per kb is higher than the ∼3 error/kb expected from 50 rounds of amplification at previously reported Taq error rates (52–54).

Next, we quantified the rates of single- and multiple-base deletions. We find that the median single-base deletion rate per position ($5.64 \times 10^{-4}$), and that this rate did not vary significantly over the positions (Mann-Whitney U, NS). We also find that multiple-base deletions occur at a similar rate as single-base deletions ($3.35 \times 10^{-4}$), and measure positional effects for where they occur. Some of this dependence can be explained by the fact that the positions of multiple-base deletions are mapped to the left-most deleted base. Thus, we expect the total number of multiple-base deletions to be highest at position one and decreasing after, since there are the most possible combinations of multiple-base deletions at that position. In addition, we measure a significant decrease in the median multiple-base deletion rate in the annealing region (positions 36-64) of our assembly (Mann-Whitney U, $p << 0.001$). Large deletions in this region would disrupt the hybridization of the initial assembly, leading to sequence drop-outs and a decrease in the measured number of deletions. We also expect the multiple deletion rate to drop towards the end of the sequence due to a "TATATAT" motif at positions 92-98. Any "TA" deletion (or other substring contained multiple times in the motif) will map to the left-most position, 92.

Finally, we quantified single-base insertions. These errors occur at median rate per position of $9.65 \times 10^{-5}$) and exhibit no positional dependence besides an outlier at position 1. An incomplete primer trimming by `BBDuk` can explain this outlier. Here, 57 of the 152 single-base insertions

are a “T,” corresponding to the last base of the primer sequence directly upstream of our first base. Without these 57 bases, the rate of single-base insertions falls closer to the expected median value. Our method is also able to detect multiple-base insertions, which occur at a median rate of $6.16{\times}10^{-6}$), but we omit them from the Figure 2A due to their rarity.

# Supplementary Figures

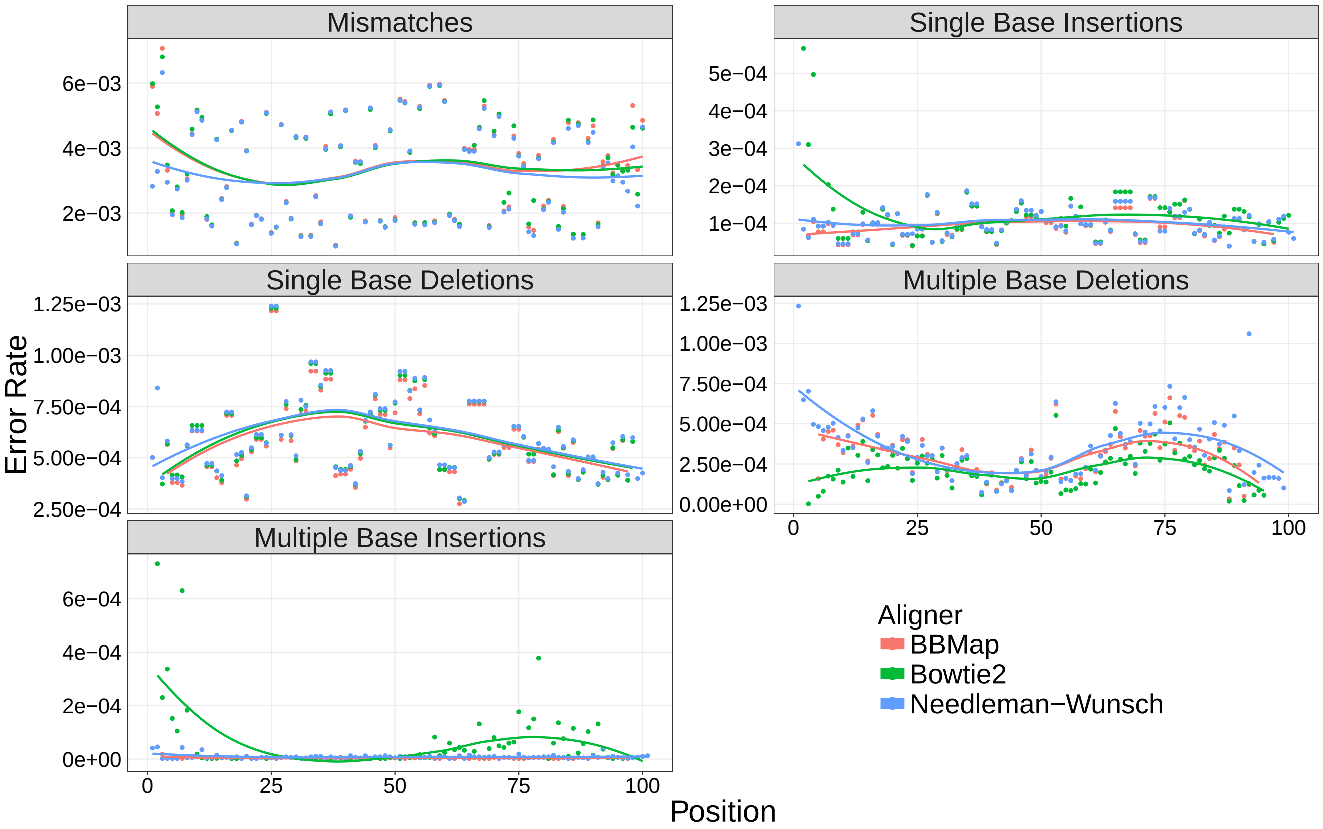


**Figure S1: Effect of read aligner on error rates.** Here we mapped reads from the standard IDT oligo with `BBMap` (red), `Bowtie2` (green), and our Needleman-Wunsch aligner (blue), and quantified the error rates with our pipeline. We see that the choice of aligner affects the resulting error rates, especially for detecting multiple-base deletions.

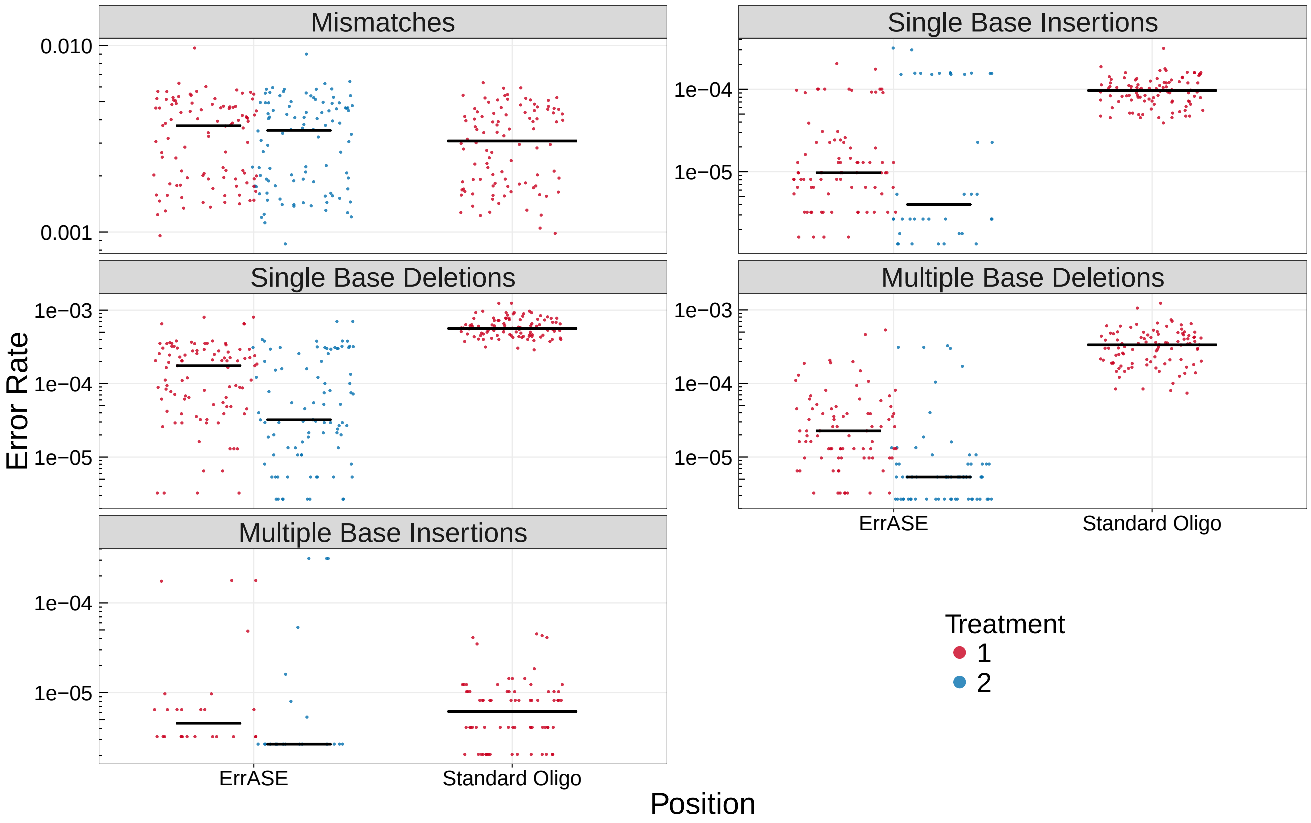


**Figure S2: Distributions of error rates per position for the standard oligo assembly before and after ErrASE treatment.** We were unable to detect a significant change between the median error rate after two treatments for mismatches. **Note:** black bar is median value.

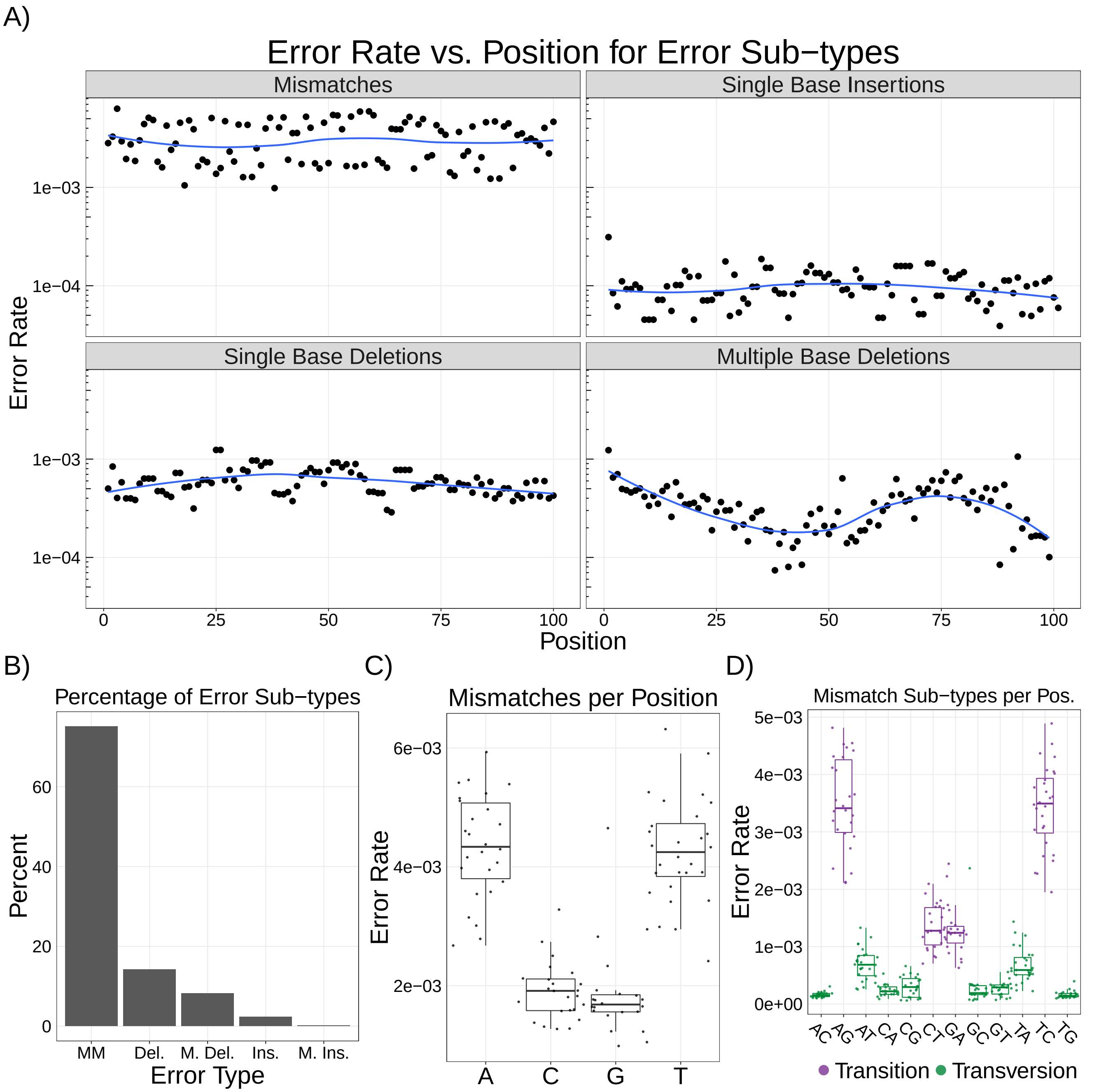


**Figure S3: In-depth analysis of error-doped assemblies.** Here, we find no significant difference in the median mismatch rate of all four bases, median transition or transversion rate, or rate of single-base deletions for each base (all tests were Mann-Whitney U, NS, Holm-corrected). **Note:** here we performed the same analysis as Figure 2 in the main text with the error-doped assembly.

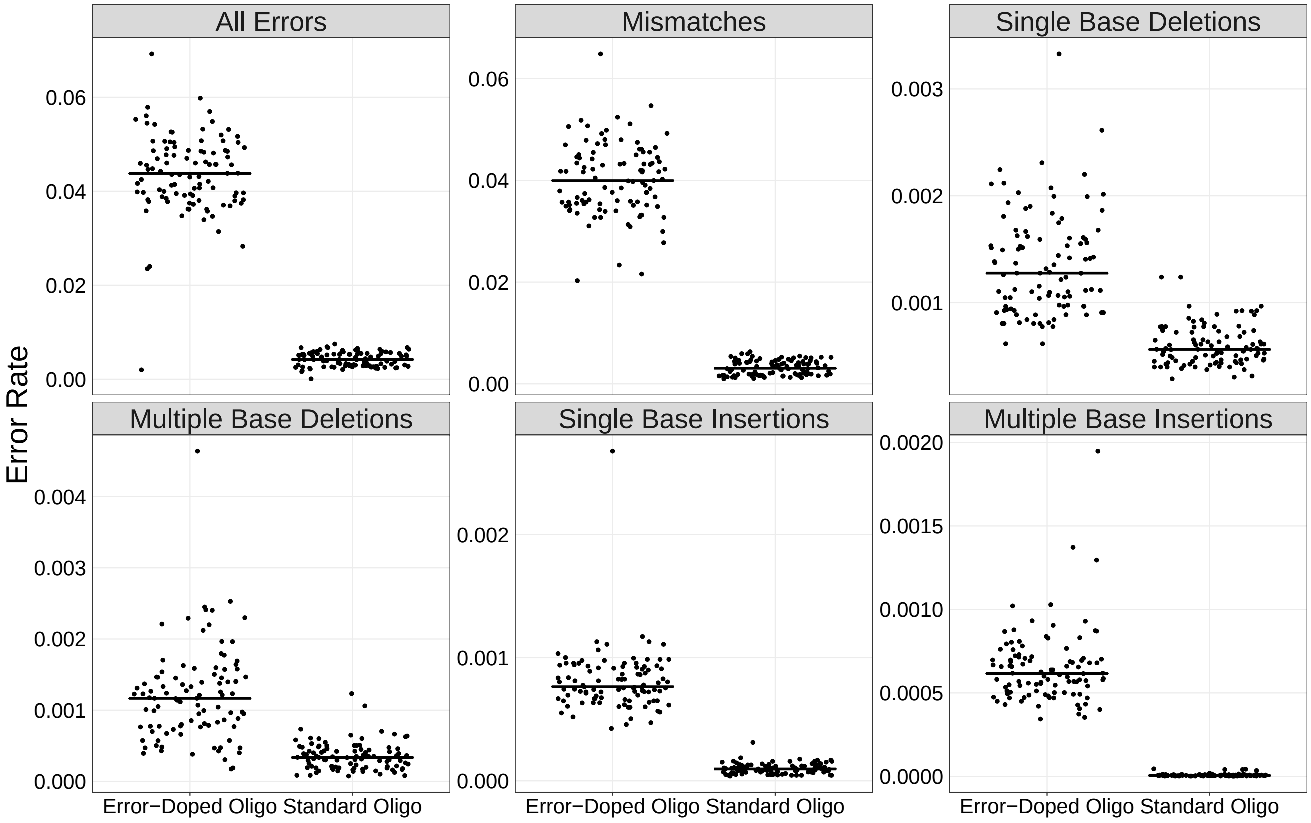


**Figure S4: Comparison of measured error rates from error-doped and standard oligos.** Here we plot the distribution of error rates per position and see that for every error sub-type the error rates are significantly higher for the error-doped oligos than those produced by the standard process (Mann-Whitney U Test, all $p << 0.001$). **Note:** Black bar is the median value.

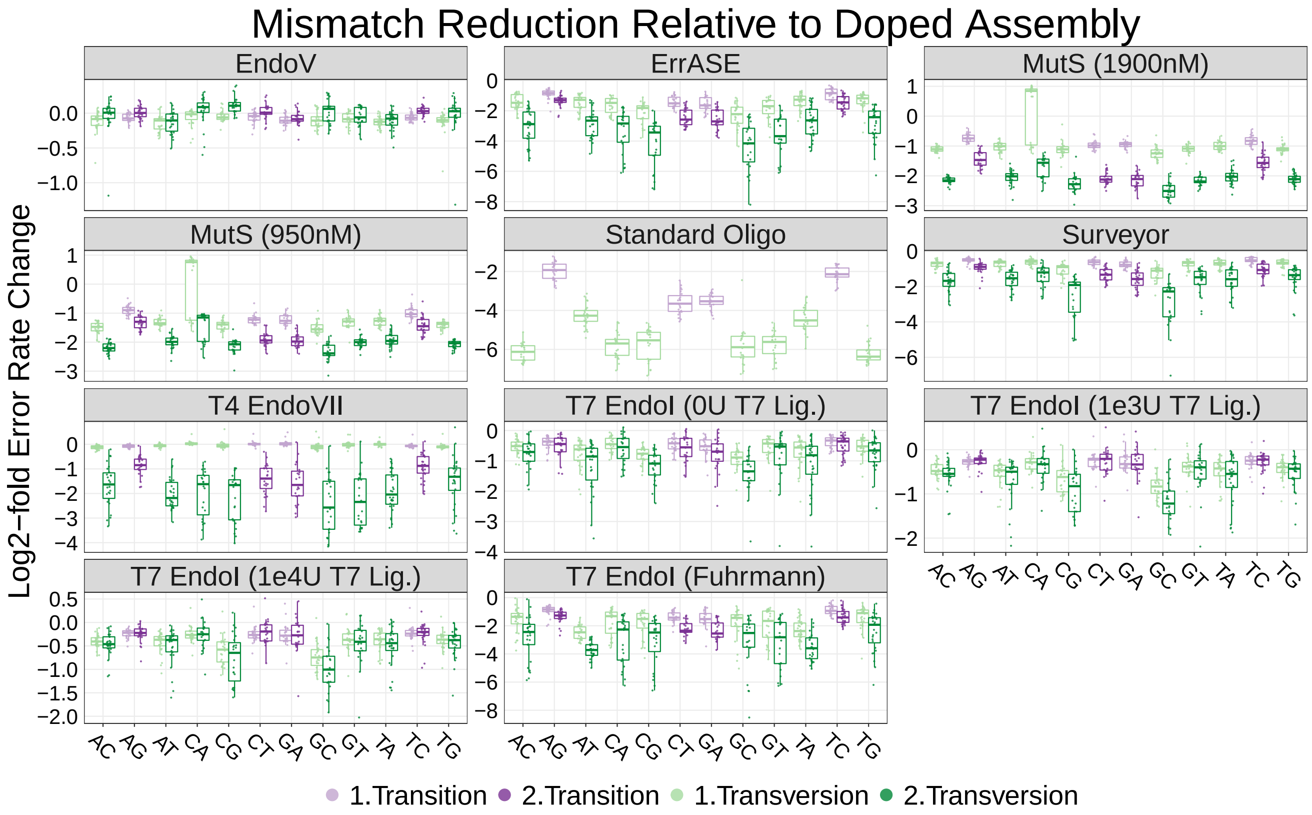


**Figure S5: Mismatch correction preferences relative to the error-doped oligo for every enzyme across two consecutive treatments.** Error rates are plotted as the $\log_2$-fold-change in error rate relative to the error-doped template. **Note:** box plots are first and third quartile for hinges, median for bar, and 1.5× the inter-quartile range for whiskers.

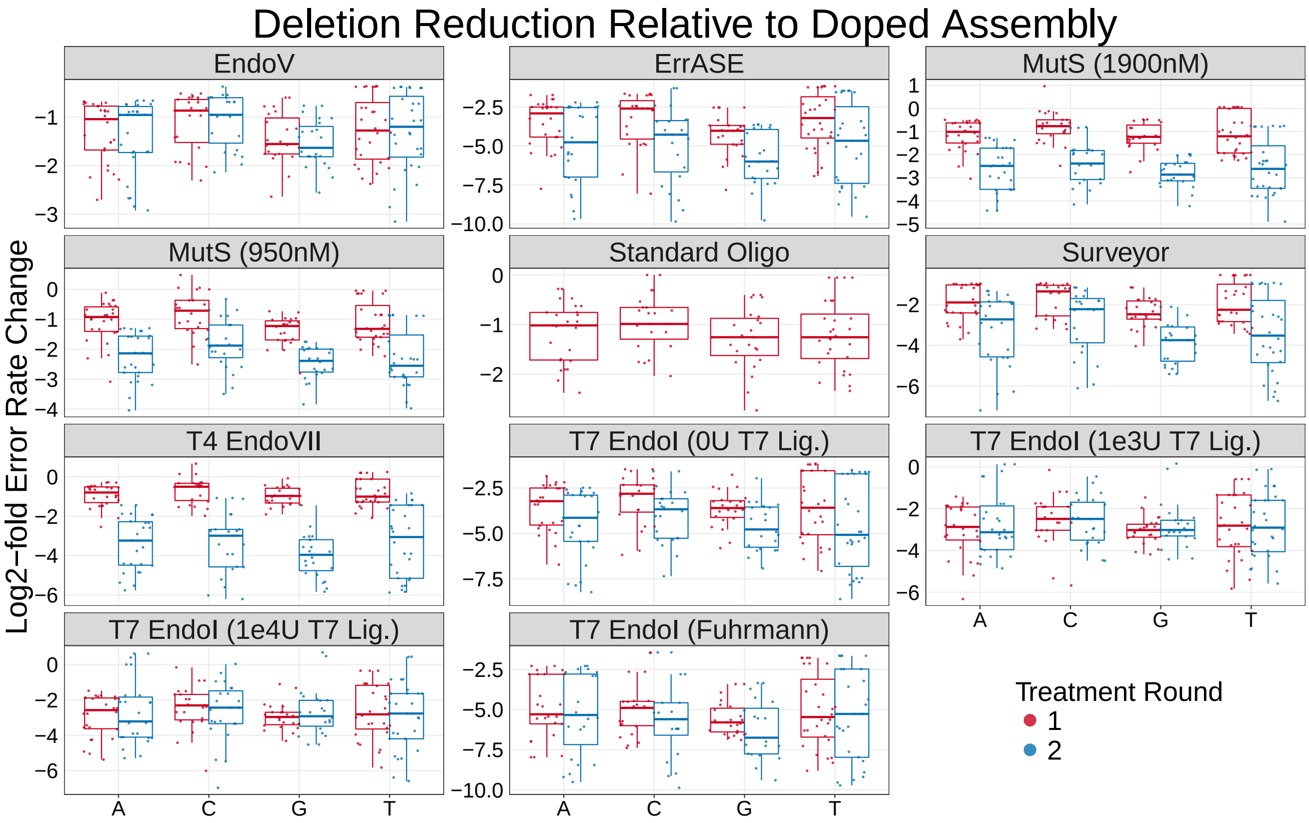


**Figure S6: Single-base deletion correction preferences relative to the error-doped oligo for every enzyme across two consecutive treatments.** Error rates are plotted as the $\log_2$-fold-change in error rate relative to the error-doped template. **Note:** box plots are first and third quartile for hinges, median for bar, and 1.5× the inter-quartile range for whiskers.

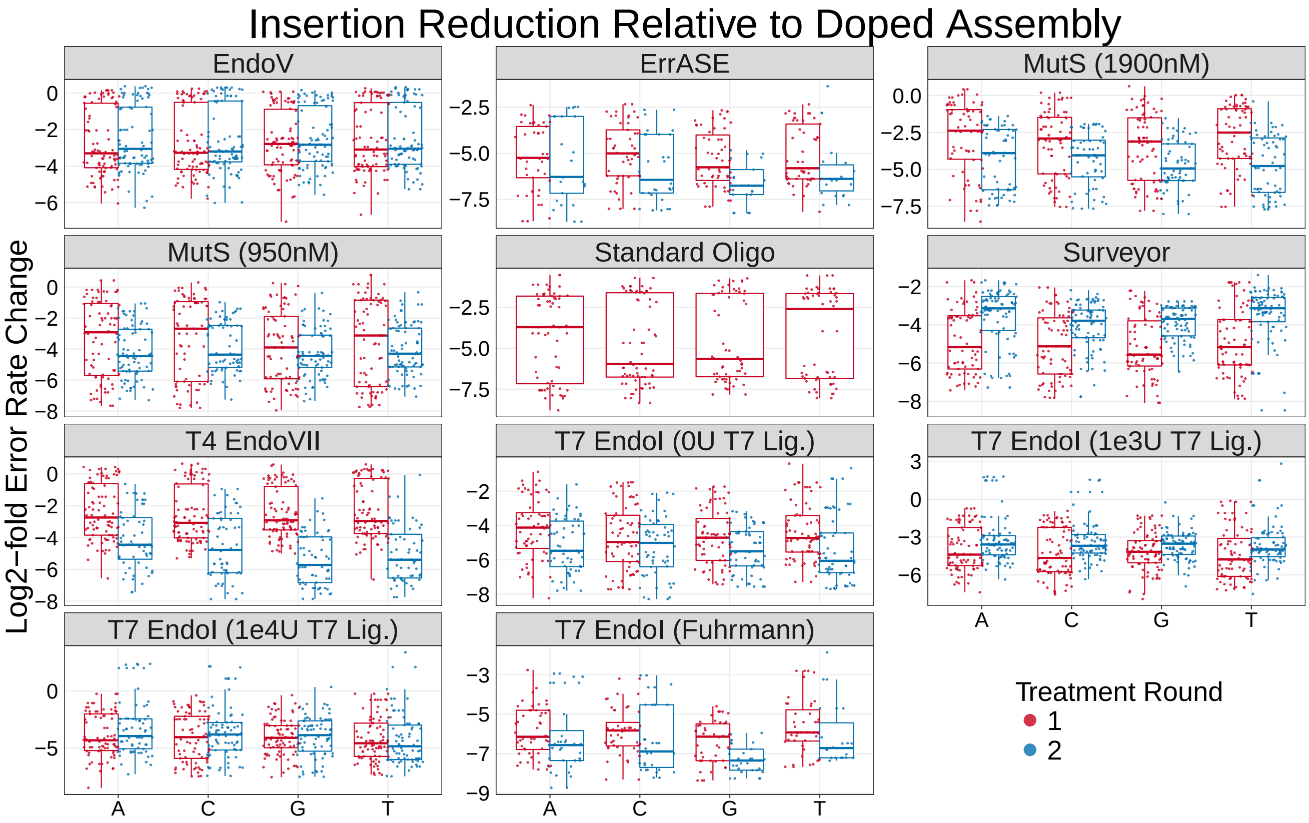


**Figure S7: Single-base insertion correction preferences relative to the error-doped oligo for every enzyme across two consecutive treatments.** Error rates are plotted as the $\log_2$-fold-change in error rate relative to the error-doped template. **Note:** box plots are first and third quartile for hinges, median for bar, and 1.5× the inter-quartile range for whiskers.

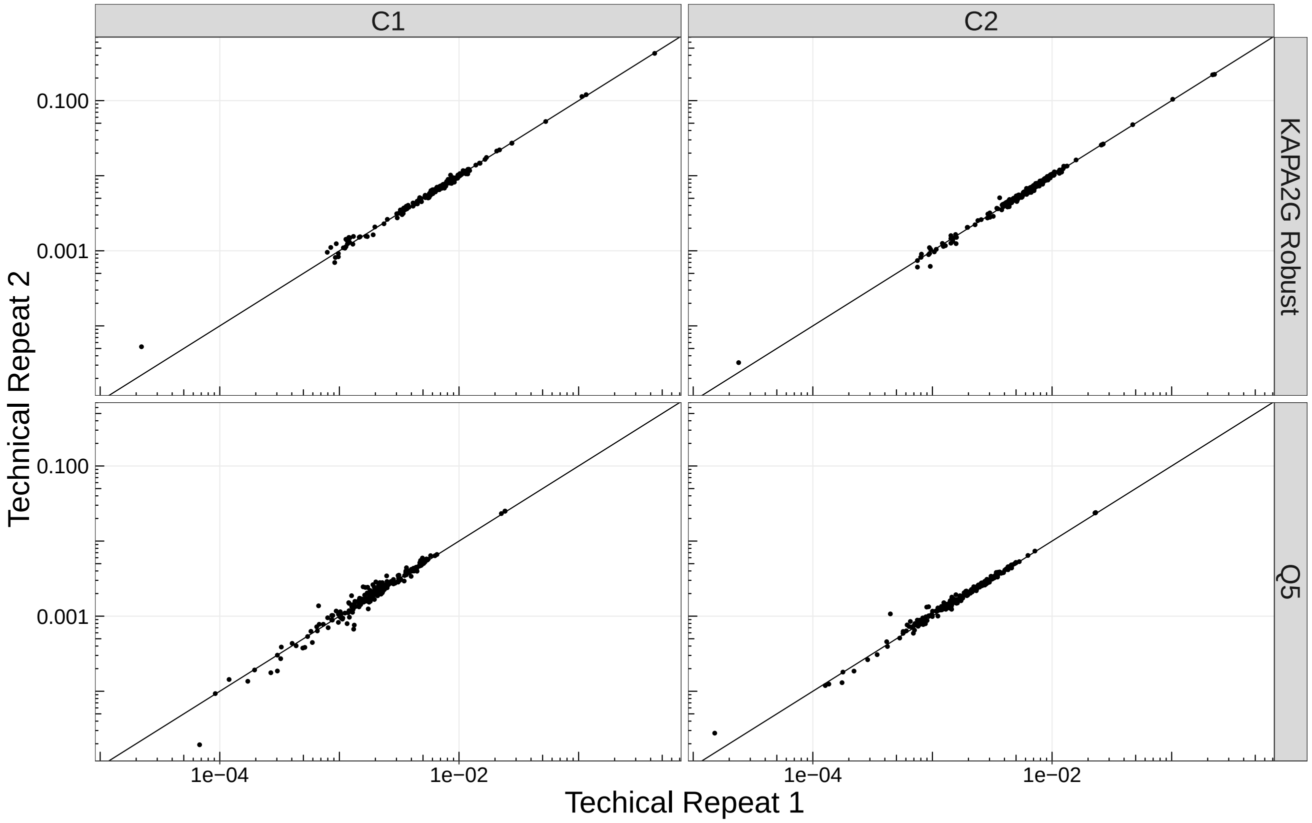


**Figure S8: Correlations between error rates for five-oligo assembly technical replicates.** We see that technical replicates are almost perfectly correlated (all $r > 0.995$), with the black line being $y = x$.

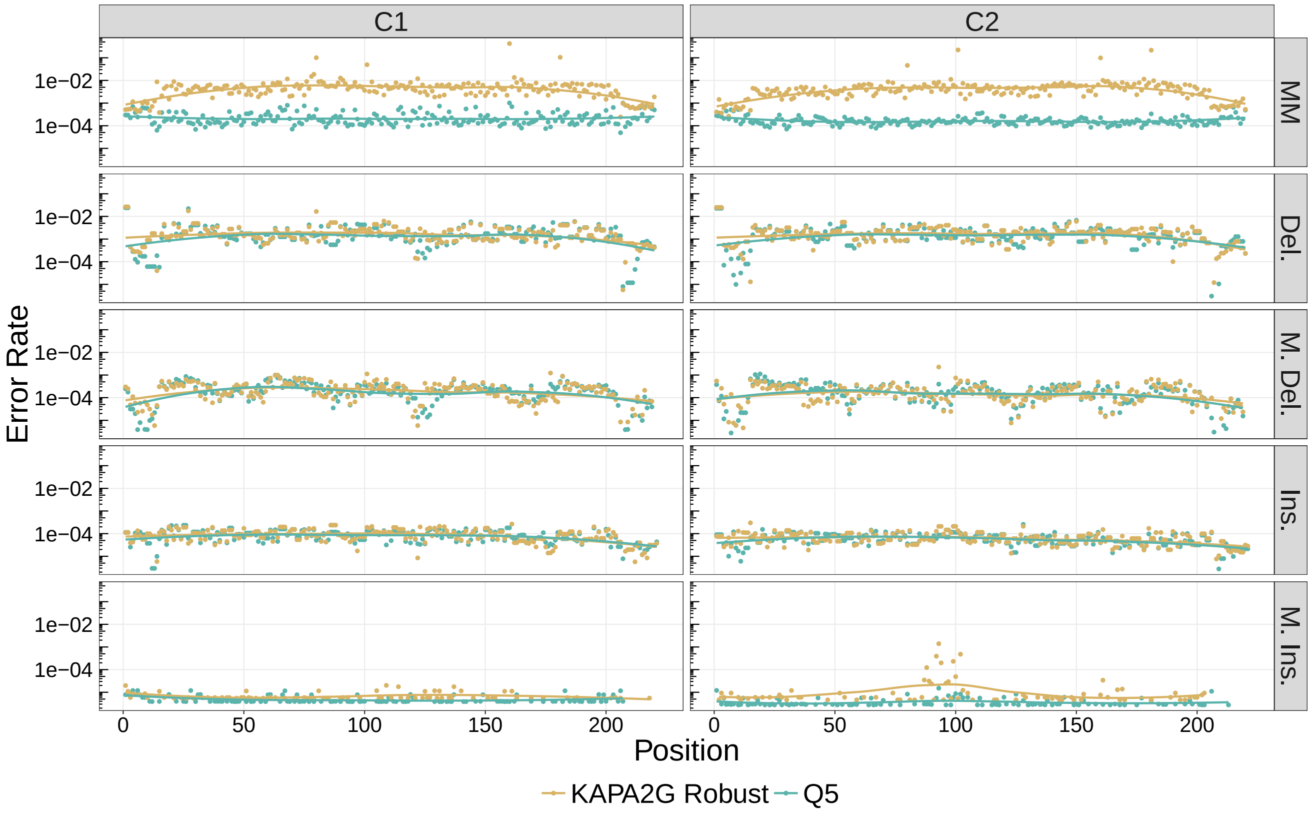


**Figure S9: Positional error rate distributions two assemblies using KAPA2G Robust and Q5 polymerase.** We see that KAPA2G Robust, a Taq-based low-fidelity polymerase, incorporates Mismatches (MM) at nearly two-orders of magnitude higher than Q5, a high-fidelity polymerase. We find that both polymerases incorporate single base deletions (Del.), multiple base deletions (M. Del.), single base insertions (Ins.), and multiple base insertions (M. Ins.) at nearly identical rates. With the exception of multiple base insertions, these trends are robust to the different sequence contexts of the two constructs. We note that KAPA2G Robust incorporates a higher number of multiple base insertions around three tandem GGA repeats, likely due to polymerase slippage.

**Table S1: Examples of where various aligners fail.** Here _ are padding for visualization, * are soft-trimming, and lower-case bases are inserts.

| Aligner: | Ideal | Needleman-Wunsch | Bowtie2 | BBMap |
|---|---|---|---|---|
| Reference: | _GCTGCCGATTTCCA... | _GCTGCCGATTTCCA... | G_CTGCCGATTTCCA... | *GCTGCCGATTTCCA... |
| Read: | aGCTGCCGATTTCCA... | aGCTGCCGATTTCCA... | aGCTGCCGATTTCCA... | *GCTGCCGATTTCCA... |
| Reference: | __GCTGCCGATTTCCA... | __GCTGCCGATTTCCA... | G__CTGCCGATTTCCA... | **GCTGCCGATTTCCA... |
| Read: | aaGCTGCCGATTTCCA... | aaGCTGCCGATTTCCA... | aaGCTGCCGATTTCCA... | **GCTGCCGATTTCCA... |
| Reference: | GCTGCCGATTTCCA... | GCTGCCGATTTCCA... | GCTGCCGATTTCCA... | GCTGCCGATTTCCA... |
| Read: | GCT___GATTTCCA... | GCT___GATTTCCA... | ___GCTGATTTCCA... | ___GCTGATTTCCA... |
| Reference: | GCTGCCGATTTCCA... | GCTGCCGATTTCCA... | GCTGCCGAT_TTCCA... | GCTGCCGATTTCCA... |
| Read: | GCTG_____TTCCA... | GCTG_____TTCCA... | ______GCTGTTCCA... | GCTG_____TTCCA... |
| Reference: | ...TGTTGTATATATCG_ | ...TGTTGTATATATCG_ | ...TGTTGTATATATC_G | ...TGTTGTATATATCG* |
| Read: | ...TGTTGTATATATCGa | ...TGTTGTATATATCGa | ...TGTTGTATATATCaG | ...TGTTGTATATATCG* |
| Reference: | ...TGTTGTATATATC__G | ...TGTTGTATATATC__G | ...TGTTGTATATATC__G | ...TGTTGTATATATCG** |
| Read: | ...TGTTGTATATATCatG | ...TGTTGTATATATCatG | ...TGTTGTATATATCatG | ...TGTTGTATATATCa** |
| Reference: | ...TGTTGTATATAT__CG | ...TGTTGTATATA__TCG | ...TGTTGTATATA__TCG | ...TGTTGTATATATCG** |
| Read: | ...TGTTGTATATATgtCG | ...TGTTGTATATATgtCG | ...TGTTGTATATATgtCG | ...TGTTGTATATATgt** |
| Reference: | ...TGTTGTATATATCG | ...TGTTGTATATATCG | ...TGTTGTATATATCG | ...TGTTGTATATATCG |
| Read: | ...TGTTGTATATA__G | ...TGTTGTATATA__G | ...TGTTGTATATAG__ | ...TGTTGTATATAG__ |
| Reference: | ...TGTTGTATATAT__CG | ...TGTTGTATATA__TCG | ...TGTTGTATATA__TCG | ...TGTTGTATATATCG** |
| Read: | ...TGTTGTATATATgtCG | ...TGTTGTATATATgtCG | ...TGTTGTATATATgtCG | ...TGTTGTATATATgt** |

**Table S2: Median error rate per position for assemblies using the error-doped oligos or the standard oligos.** We measure significant (Mann-Whitney U, $p << 0.001$) differences between the median error rates of the error-doped and standard oligos for all error sub-types.

| Type | Error-Doped Oligo | Standard Oligo |
|---:|---:|---:|
| All Errors | $4.38 \times 10^{-2}$ | $4.18 \times 10^{-3}$ |
| Mismatches | $3.99 \times 10^{-2}$ | $3.08 \times 10^{-3}$ |
| Single Base Deletions | $1.28 \times 10^{-3}$ | $5.64 \times 10^{-4}$ |
| Multiple Base Deletions | $1.17 \times 10^{-3}$ | $3.35 \times 10^{-4}$ |
| Single Base Insertions | $7.64 \times 10^{-4}$ | $9.65 \times 10^{-5}$ |
| Multiple Base Insertions | $6.16 \times 10^{-4}$ | $6.16 \times 10^{-6}$ |
